# First representative complete mitochondrial genome of the *Taphozous melanopogon* Temminck, 1841 (Chiroptera: Emballonuridae) from China

**DOI:** 10.1080/23802359.2022.2081940

**Published:** 2022-07-18

**Authors:** Yan-nan Li, Wen-hua Yu, Sanjan Thapa, Yi Wu

**Affiliations:** Key Laboratory of Conservation and Application in Biodiversity of South China, School of Life Sciences, Guangzhou University, Guangzhou, PR China

**Keywords:** *Taphozous melanopogon*, Chiroptera, Emballonuridea, mitochondrial genome

## Abstract

In this study, we present the first representative complete *Taphozous melanopogon* mitochondrial genome from China. Its mitochondrial genome was assembled and annotated using MitoZ. The genome is a circular molecule of 16,566 bp in length, including 22 transfer RNA genes, 2 ribosomal RNA genes, 13 protein-coding genes, and a control region. Although maximum-likelihood and Bayesian inference phylogenetic trees indicate that the super family Emballonuridea forms a sister taxon with Noctilionidea instead of Vespertilionidea, mitochondrial genes provide only part of the phylogenetic information, and phylogenetic inferences utilizing nuclear genes are needed in future toward resolving phylogenetic relationship among Vespertilionidea, Noctilionidea, and Emballonuridea.

Black-beard Tomb Bat *Taphozous melanopogon* Temminck, 1841 belonging to the superfamily Emballonuridea. It is one of the widespread tomb bat species in Asia, including China, Indonesia, Burma, Thailand, and Vietnam (Kitchener et al. 1993; Wilson and Mittermeier 2019). In China, it occurs in tropical and subtropical regions including Guangdong, Guangxi, Yunnan, Guizhou, Hainan, Macao, Beijing, and Hong Kong (Jiang et al. 2017). Nowadays, the phylogenetic history of Emballonuridea remains a conflict among Vespertilionidea and Noctilionidea (Teeling et al. 2000; Teeling et al. 2002; Van den Bussche and Hoofer 2004; Eick et al. 2005; Teeling et al. 2005; Miller-Butterworth et al. 2007; Amador et al. 2018).

In this study, a male individual of *Taphozous melanopogon* (Voucher No. GZHU 15063) was sampled in a cave near Longmen Town, Guangdong Province, China (23.59° N, 114.29° E) in 2015. The person in charge of the collection: Yi Wu (email: wuyizhouq@263.net). The specimen is presently deposited at Key Laboratory of Conservation and Application in Biodiversity of South China, School of Life Sciences, Guangzhou University (contact email: wuyizhouq@263.net). Permission for field surveys and sampling was granted by the Forestry Administration of Guangdong Province, China. The identification of *Taphozous melanopogon* was confirmed by phylogenetic analyses using datasets comprising *cyt*b and *cox*1 as well as morphological examinations (Corbet and Hill 1992; Dengis 1996; Colket and Wilson 1998; Bates et al. 2000). Total genome was extracted from liver tissue using MiniBEST Universal Genomic DNA Extraction kit (TAKARA, Dalian) and was further sequenced paired-end using MGISEQ-2000 sequencing platforms, following a PE150 protocol. Based upon ∼5GB data a complete mitochondrial genome was assembled and annotated via MitoZ v2.4 which is specialized for mitochondrial genome (Meng et al. 2019).

Our study represents the first mitochondrial report of genus *Taphozous.* Mitochondrial genome of the *Taphozous melanopogon* is 16,566 bp in length (Genbank accession No. MZ286363), containing 13 protein-coding genes, 22 *t*RNA genes, 2 *r*RNA genes, and a control region. Among the 13 protein-coding genes, *atp*8 and *atp*6 were overlapped by 43 bp, *nad*4L and *nad*4 were overlapped by 7 bp. Most start codon of the protein-coding genes is ATG, except for *nad*2(ATT) and *nad*3 (ATT), *nad*5 (ATA). Termination codon of eight protein-coding genes were TAA (*atp*8, *atp*6, *cox*1, *cox*2, *nad*1, *nad*4L*, nad*5, and *nad*6), while the rest genes were different, including, *nad*2 (TAG), *nad*3(TAG). Three genes end with an incomplete stop codon TA- (*cox*3) and T– (*cyt*b, *nad*4*)*, which can be modified by the polyadenylation after transcript processing (Ojala et al. 1981). *rrn*S gene and *rrn*L were separated by *trn*V, lengths of them were 970 bp and 1561 bp, respectively. Control region is between the *trn*F and the *trn*P, and it is 1138 bp in length.

In phylogenetic analyses, we covered the sequence of representatives from Emballonuridea, Noctilionidea, Vespertilionidea, Rhinolophoidea, and Pteropodidae. Yangochiroptera lineages (Rhinolophoidea and Pteropodidae) were set as outgroup (Figure 1). The 37 genes were extracted for phylogenetic inference using by PhyloSuite v1.1.2 (Zhang et al. 2020). While the mitochondrial control region was eliminated because of its high variability. We aligned our sequence matrixes using MUSCLE (Edgar 2004) and optimized the alignments of protein-coding genes using MACSE v2 (Ranwez et al. 2018). Conserved blocks were further identified of using Gblock (Talavera and Castresana 2007). ModelFinder was adopted to determinate optimal model for each gene partition (Kalyaanamoorthy et al. 2017). The maximum-likelihood phylogenetic trees were inferred using IQ-Tree v2.0.3 with 1000 bootstraps setting (Minh et al. 2020), Bayesian phylogenetic inference was using MrBayes v3.2.6. Monte Carlo–Monte Carlo chains were simultaneously run for 10 million generations, with sampling conducted every 1000 generations. The confidence values of the tree are presented as Bayesian posterior probabilities. (Ronquist et al. 2012). Both phylogenies depicted Emballonuridea as sister taxon to Noctilionidea (Figure 1). Given the fact that mitochondrial genes provide only part of the phylogenetic information. Discordance between mitochondrial genes and nuclear genes in animals (Toews and Brelsford 2012), phylogenetic inference utilizing nuclear genes are needed in future toward resolving phylogenetic relationship among Vespertilionidea, Noctilionidea and Emballonuridea.

**Figure 1.**
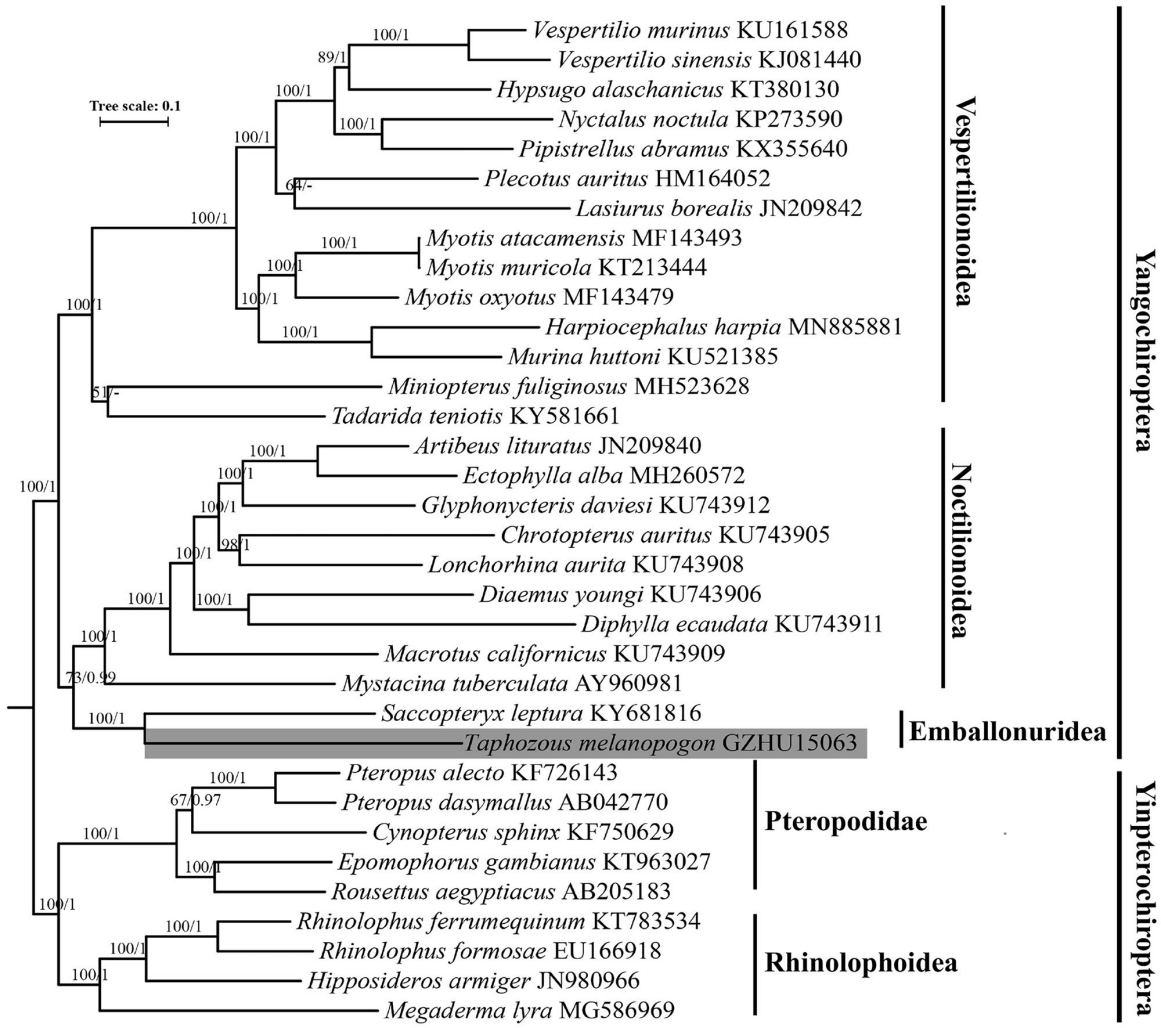
Maximum likelihood (ML) and Bayesian inference (BI) phylogenetic trees inferred from 37 mitochondrial genes, node support values are shown above branches as ML bootstrap values (before slash) and Bayesian posterior probabilities (after slash).

## Author contributions

Yi Wu and Wen-hua Yu designed the study; Yan-nan Li preformed phylogenetic analyses; Sanjan Thapa revised the manuscript.

## Data Availability

The data that support the findings of this study are openly available in NCBI at https://www.ncbi.nlm.nih.gov/nuccore/MZ286363, reference number MZ286363. The associated BioProject, BioSample and SRA numbers are PRJNA730371, SAMN19236524 and SRR15311579 respectively.
